# An Overview of Human T-Lymphotropic Virus Type 1 Lung Injury

**DOI:** 10.3389/fimmu.2022.914498

**Published:** 2022-07-01

**Authors:** Ápio Ricardo Nazareth Dias, Luiz Fábio Magno Falcão, Juarez Antônio Simões Quaresma

**Affiliations:** ^1^ Health and Biologic Center, State University of Pará, Belém, Brazil; ^2^ Tropical Medicine Centre, Federal University of Pará, Belém, Brazil; ^3^ University of São Paulo, São Paulo, Brazil

**Keywords:** HTLV-1, HAM/TSP, chest CT, pulmonary disease, pulmonary function

## Abstract

Previous studies have demonstrated the development of pulmonary impairment in individuals infected with human T-lymphotropic virus type 1 (HTLV-1). Complications, such as alveolitis and bronchiectasis, were found in individuals who developed tropical spastic paraparesis/HTLV-1-associated myelopathy (TSP-HAM) due to chronic inflammation. These patients exhibited increased levels of lymphocytes (CD4+ and CD25+), cytokines (IL-2, IL-12, and IFN-γ), inflammatory chemokines (MIP-1α and IP-10), and cell adhesion molecules (ICAM-1) in the bronchoalveolar lavage fluid, with the result of chronic inflammation and lung injury. The main lesions observed at Chest high-resolution computed tomography were centrilobular nodules, parenchymal bands, lung cysts, bronchiectasis, ground-glass opacity, mosaic attenuation, and pleural thickening. It can lead to progressive changes in pulmonary function with the development of restrictive and obstructive diseases. Recent studies suggest a causal relationship between HTLV-1 and pulmonary diseases, with intensification of lesions and progressive decrease in pulmonary function. This summary updates a previous publication and addresses the general lack of knowledge regarding the relationship between TSP-HAM and pulmonary disease, providing direction for future work and the management of these individuals.

## Introduction

Human T-lymphotropic virus type 1 (HTLV-1) is a retrovirus with an incidence of approximately 20 million worldwide, with a higher prevalence in Africa, Japan, and America (1). In Latin America, Brazil has a high prevalence, mainly in the states of Maranhão, Bahia and Pará (2, 3). The virus is the etiological agent of tropical spastic paraparesis/HTLV-1-associated myelopathy (TSP-HAM) and adult T-cell lymphoma (ATL) (4).

**Figure 1:**
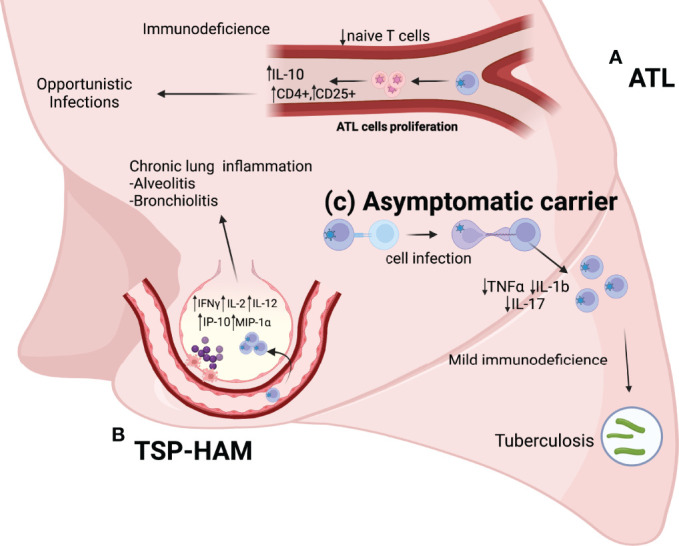
**(A)** ATL: ATL cell proliferation leads to a reduction in the number of naive T cells, a increase in the number of T reg cells (CD4+ and CD25+), and increased expression of anti-inflammatory cytokines (IL-10). Pneumopathies in these individuals are mainly caused by opportunistic diseases. **(B)** TSP-HAM: The high expression of inflammatory cytokines (IFNγ, IL-2, and IL-12) and chemokines (IP-10 and MIP-1α) results in an exacerbated immune response and chronic lung inflammation (Alveolitis and bronchiolitis). **(C)** HTLV-1 asymptomatic carriers: The low expression of TNFα, IL-17, and IL-1b causes mild immunodeficiency in these individuals, with a higher risk of infection by *Mycobacterium tuberculosis*.

There is a relationship between HTLV-1 and pulmonary diseases in individuals with TSP/HAM, these individuals exhibit pulmonary diseases with characteristics of lymphocytic inflammatory infiltrates (5–8). Individuals with ATL develop pneumopathies caused by opportunistic infections due to ATL cell proliferation, which leads to a low expression of naive T cells, increased expression of FoxP3+ and interleukin-10 (IL-10), and an increased number of Treg cells (CD4+ and CD25+), which suggests the development of immunodeficiency (9). Furthermore, HTLV-1 carriers, because of a mild immunodeficiency characterized by a low expression of IL-1b and IL-17 interleukins (10) have a higher risk of infection with *Mycobacterium tuberculosis * (11, 12), high mortality rates, and an increased likelihood of hospitalization for pulmonary tuberculosis (13) (**Figure 1**).

TSP-HAM individuals have a major risk to development of lung injuries, being the major radiological findings bronchiectasis, centrilobular nodules, and ground-glass opacities (14–16); lesions are attributable to chronic inflammation resulting from the effects of the virus *in situ* (17–20). Lung inflammation may be the causal agent of lung volume obstruction, flow limitation, and the development of restrictive and obstructive lung diseases in TSP-HAM patients (17, 19, 21).

Recent publications, including a systematic review and a cohort study developed by our research group, have suggested a causal relationship between HTLV-1 infection, the development of lung injury (20, 22), and the evolution of lung disease in HTLV-1 infected individuals (23). This scientific literature review aims to update our previous publication (19) with these recent findings on HTLV-1 pulmonary disease and the existing lack of knowledge regarding the effects of this infection on the respiratory system.

## Pathophysiology of TSP-Ham Related Pulmonary Disease

### Immune Response

The chronic pulmonary inflammation in TSP-HAM individuals can be caused by an exacerbated immune response. The elevation of T lymphocytes in the bronchoalveolar lavage fluid (BALF) of HTLV-1 individuals pulmonary involvement is characterized by a cytokine storm, with high expression of soluble IL-2 receptors (IL-2R), as well as, interleukins (IL-2, IL-12), and interferon (IFN-γ) (8, 24, 25).

A selective T-cells infiltration occurs in the lungs, with an accumulation of HTLV-1-specific CD8+ T cells in BALF, and the occurrence of specific immune responses in lung tissues (7, 26). The high-expression of lymphocites, and its interaction with cytokines (IL-2, IL-12 and IFN-y) and chemokines (MIP-1a and IP-10) leads to chronic pulmonary inflammation and lung injury (25, 27). It is known that HTLV-1 infection induces an abnormal frequency and phenotype of FoxP3^+^CD4^+^T cells (28). The higher expression of *Foxp3 *mRNA in the BALF of patients with HTLV-1-related lung diseases suggests the involvement of regulatory T cells in the pathogenesis of lung injuries (8).

### Chronic Inflammation

TSP-HAM individuals exhibit alveolitis, a high proviral load (29), and increased levels of cytokines and inflammatory chemokines in the BALF in comparison to asymptomatic carriers (8, 25, 27, 30). The lymphocytosis in the lungs results in a higher expression of proinflammatory cytokines (31–33).

**Figure 2:**
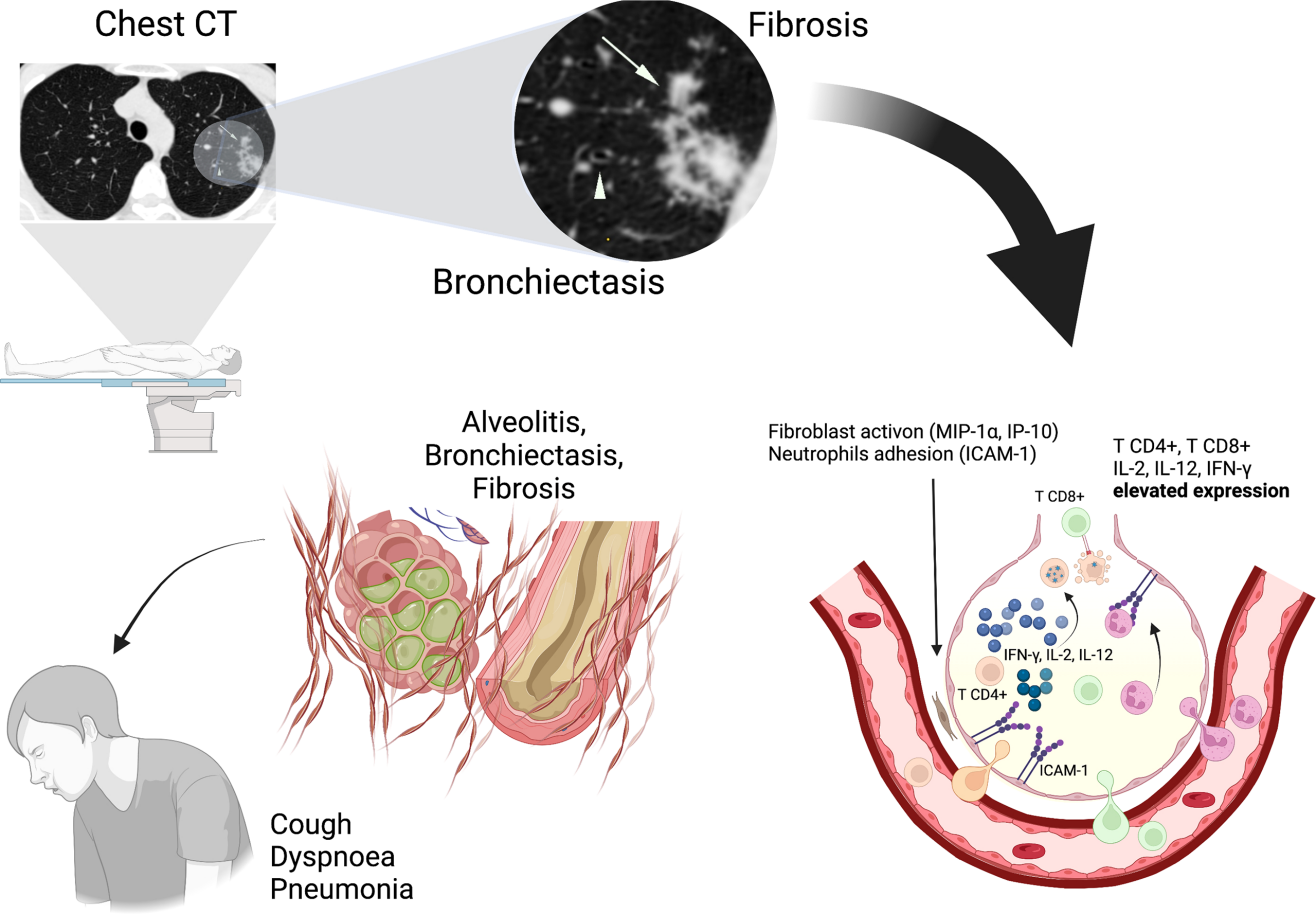
Pulmonary inflammation involves the interaction between cytokines (IL-2, IL-12, and IFN-γ) and chemokines (MIP-1α and IP-10) with HTLV-1 infected CD4+ T cells resulting in lung injuries (alveolitis, bronchiectasis). ICAM-1 facilitates the adhesion of neutrophils and potentiates the chronic inflammation. Chest CT imaging shows Bronchiectasis (Arrowhead) that indicates enlargement and deformation of airway and Centrilobular nodule (Arrows) that indicates Fibrosis, bronchiolitis and alveolitis at sites of injury. These alterations determine a chronic evolution with presence of symptoms (Cough, Dyspnoea, Pneumonia).

Lymphocytosis and the presence of HTLV-1 provirus in the BALF (7), elevated levels of macrophage inflammatory protein (MIP-1α), interferon g-induced protein kDa (IP-10), and chemokines are linked with the activation and recruitment of inflammatory cells (30, 34). The pulmonary epithelium expresses intercellular adhesion molecule-1 (ICAM-1), a chemokine that facilitates the adhesion of neutrophils to cells of the respiratory epithelium (30, 34) and induces lung tissue injury and chronic inflammation *in situ* (16) (**Figure 2**).

### Lung Injury

The development of lung injuries, mainly bronchiectasis and centrilobular nodules are related to alveolitis and bronchiolitis (27, 35). These lung injuries cause scarring in the lung tissue and fibrosis, which can induce traction bronchiectasis in a cycle of chronic lung injury (17). TSP-HAM individuals have a bronchiectasis relative risk of 8.4 (95% CI 2.7-26.1, p = 0.0002) in comparison to asymptomatic carriers and other HTLV-1 related diseases (16).

Other imaging findings reinforce the existence of a causal relationship between pulmonary diseases and HTLV-1; the centrilobular nodules indicate peripheral bronchiolitis and alveolitis at sites of injury, probably due to lymphocytosis (7). Ground-glass opacity is characteristic of pneumonia and has a higher prevalence among patients with HTLV-1 than in the general population (15).

## CT Findings

Chest high-resolution computed tomography is the gold standard method to observe lung injuries. Previous studies have shown that the characteristic lesions observed in HTLV-1 infected individuals are bronchiectasis (8, 16, 17, 24, 25, 27), bronchiectasis is characterized by bronchial dilatation (36). Other lung injuries, such as centrilobular nodules, ground-glass opacity, pleural thickening, and parenchymal bands, were also found (14, 15, 17, 36) (**Figure 2**).

The studies about HTLV-1 related lung diseases shows that these abnormal CT findings are more common in TSP-HAM individuals than asymptomatic carriers (16, 17, 23), their higher frequency of lung injury can be explained by their major *in situ* inflammatory processes (8, 17, 24, 25, 27) and is associated with high HTLV-1 proviral load (37, 38). These individuals also exhibit three or more lesions types, and a combination between bronchiectasis and other lesions in HRCT, such as pleural thickening, parenchymal bands, interlobular septum thickening, centrilobular nodules, and parenchymal bands (17). A follow-up study shows the intensification of these lesions, and an increase in the frequency of four types: ground-glass opacity, bronchiectasis, centrilobular nodules, and pleural thickening between TSP-HAM individuals previous evaluated (23).

## Pulmonary Function

Individuals with TSP-HAM can develop changes in pulmonary function, due to pulmonary inflammation and lung lesions, which may progress to obstructive or restrictive lung disease (17, 21). An analysis of pulmonary function in these individuals showed a reduction in vital capacity (VC) and forced expiratory volume in one second (FEV1), these alterations are related to restrictive lung disease, and airway obstruction, respectively (17).

Other findings were a reduction in peak expiratory flow, which is very sensitive in most diseases that affect the lungs, alteration in the 50% Forced expiration flow (FEF50%), common alteration in the early stages of obstructive lung disease, and reduction in 25-75% Final Expiratory Flow (FEF 25-75), that is linked to histological changes in the peripheral airways and obstruction (17, 21, 23).

Finally, a reduction in maximum voluntary ventilation (MVV) was observed (17, 21, 23). Changes in MVV may be present both in diseases that affect the lungs and in adverse conditions that alter the mobility of the rib cage (39). HTLV-1 individuals tend to have decreased lung values and this may be related to the development of motor changes related to myelopathy associated with TSP-HAM (17).

The downward trend in VC, FVC, and FEV1, with the maintenance of a normal ratio of forced expiratory volume in one second to forced vital capacity (FEV1/FVC) values, may indicate the development of restrictive lung disease; however, this restriction must be confirmed by measuring lung values and documenting total lung capacity below normal limits (40). The MVV measure is related to the level of physical activity in daily life and is applied to individuals with chronic obstructive pulmonary disease (41). Abnormal CT findings, with airway and lung scarring lesions observed in HTLV-1 individuals, associated with the low mobility that affects patients with TSP-HAM may play a key role in pulmonary function changes (17).

A follow-up study showed a decrease in lung function related to lung injuries observed by chest CT; the patient group with lung injury showed a tendency of decline in VC, FVC, FEV1, FEF25-75%, and MVV values (23). As shown in previous studies, lung injury and altered lung function are more common in TSP-HAM individuals (17, 21), with a major degree of lung involvement among those who developed TSP-HAM. It is possible that bronchiectasis and pleural thickening play key roles in the development of obstructive and restrictive lung disease, respectively (17).

## Future Directions

The studies with Chest CT imaging shows that lung lesions are more common in TSP-HAM patients than asymptomatic individuals, suggesting that lesions at the pulmonary level follow the systemic inflammatory process. HTLV-1 infection is a systemic inflammatory disease characterized by chronic evolution. Observational studies conducted on these individuals do not allow for the determination of the pathophysiological mechanisms and their links to specific clinical presentations of patients infected with HTLV-1.

The development of lung lesions in HTLV-1 infected individuals has been described in several studies, but some points, such as the actual mechanism of action of the virus in the pulmonary system, the role of epigenetic factors and inflammatory imbalance in lung injury, and the death rate among those infected, remain unclear. These studies have a limited scope and describe only isolated clinical cases. They do not answer the question about the evolution and physiopathology of HTLV-1-related pulmonary disease.

There are a few prospective studies, such as follow-up and case-control studies, but they suggest a progressive characteristic of HTLV-1 pulmonary disease, and more studies are necessary to better understand the mechanisms of pulmonary involvement. Screening of these patients is very important to show the evolution of chronic inflammation at the pulmonary level, parenchymal lesions, and the development of new lung lesions in individuals with TSP-HAM. Periodic pulmonary evaluation is needed to improve the clinical management of these individuals. This review intends to update a review previously published by our research group, contributing to providing directions for future investigations.

## Author Contributions

AD, LF, and JQ contributed to conception and design of the study. AD, and LF wrote the sections of the manuscript. All authors contributed to manuscript revision, read, and approved the submitted version.

## Conflict of Interest

The authors declare that the research was conducted in the absence of any commercial or financial relationships that could be construed as a potential conflict of interest.

## Publisher’s Note

All claims expressed in this article are solely those of the authors and do not necessarily represent those of their affiliated organizations, or those of the publisher, the editors and the reviewers. Any product that may be evaluated in this article, or claim that may be made by its manufacturer, is not guaranteed or endorsed by the publisher.
